# Sex Differences in Incident Stage 3 Chronic Kidney Disease: A Narrative Review

**DOI:** 10.7759/cureus.113686

**Published:** 2026-07-30

**Authors:** Khudheeja A Ahmed, Anh Hoang, Ibrahim Mohsin, Sumera Ahmed

**Affiliations:** 1 College of Osteopathic Medicine, Touro University California, Vallejo, USA; 2 Department of Medicine, Touro College of Osteopathic Medicine, Vallejo, USA; 3 Department of Nephrology, Kaaj Healthcare, San Jose, USA; 4 Department of Diabetology, Touro College of Osteopathic Medicine, Vallejo, USA

**Keywords:** egfr less than 60, gender, new-onset ckd, sex differences, stage 3 ckd

## Abstract

Chronic kidney disease (CKD) progression has been shown to differ by sex, although findings across studies remain inconsistent. This narrative review examines evidence on sex differences in the development of stage 3 CKD, defined as incident estimated glomerular filtration rate (eGFR) <60 mL/min/1.73 m². A narrative literature search of PubMed was conducted to identify observational cohort studies evaluating sex-specific risk in CKD development. Findings varied by population characteristics and outcome definitions. Evidence regarding sex differences in incident stage 3 CKD is mixed, with some findings suggesting greater risk among women and others identifying male sex as a potential risk factor. Additionally, metabolic and lifestyle risk factors demonstrated sex-specific associations, with some exposures showing stronger effects in men or differing based on whether CKD was defined by eGFR decline or proteinuria. Overall, sex differences in CKD development appear to be influenced by methodological variability and population differences. Further research using standardized definitions and sex-stratified analyses is needed to clarify underlying biological and clinical mechanisms and inform sex-specific risk assessment and monitoring.

## Introduction and background

Chronic kidney disease (CKD) progression is a serious public health issue. Identifying individuals at risk of progressing to stage 3 CKD is critical for prompt intervention and improved long-term outcomes. Most large-scale studies do not monitor the transition between CKD states over time using repeated measurements for at least three months [1-3]. Consequently, this review adopts the estimated glomerular filtration rate (eGFR) less than 60 mL/min/1.73 m² as the standard criterion for defining progression into CKD stage 3. 

Recent research increasingly examines sex-dependent differences in CKD progression. According to Kidney Disease: Improving Global Outcomes (KDIGO), physiologic and genetic factors, including body composition, and gender-related factors, including social roles and access to medical care, influence the development of CKD and treatment response [4]. Despite increased attention to sex-stratified CKD outcomes, the evidence is inconsistent and difficult to compare across studies [1,2]. This variation is partly attributable to varying outcome definitions, differences in albuminuria measurements, and frequent reliance on single creatinine-based eGFR measurements rather than persistent abnormalities [3,5].

Diabetes mellitus is a primary contributor to kidney failure; therefore, examining individuals with diabetes provides insight into sex differences in CKD risk [6]. In a cohort of adults diagnosed with diabetes and preserved kidney function, Yu et al. found that female patients displayed a higher likelihood of developing CKD than men when accounting for mortality as a competing risk [7]. This finding contrasts with earlier studies, which often identified higher CKD risk in men but did not consider competing risk [1,2,8]. Failure to account for sex differences in mortality rates may bias such comparisons [7]. The results indicate that both biological factors and methodological methods can affect observed sex differences [1,2,7,8].

In addition to diabetes-specific cohorts, large population studies show that CKD risk patterns for men and women depend on how exposure and outcome are defined. Miyakoshi et al. identified that male sex was associated with a greater likelihood of CKD in a large longitudinal health screening cohort, but they did not provide separate results for men and women [8]. In contrast, Lee et al. developed sex-specific risk prediction models for incident eGFR <60 in over 11 million adults and found that CKD incidence rates and certain predictors differed between men and women, indicating sex-specific risk profiles even when using the same eGFR threshold [1]. Similarly, Park et al. studied alcohol use and CKD in over 14 million adults and observed that sex-specific differences are most pronounced for proteinuria outcomes [2]. These findings show that sex patterns may differ depending on whether CKD is defined by proteinuria or filtration measures [2]. 

Emerging evidence indicates that metabolic factors may affect CKD risk differently in men and women [9]. Kim et al. investigated longitudinal changes in the triglyceride-glucose (TyG) index, a standard marker for insulin resistance, and found that increasing TyG index predicted incident CKD in men but not in women [9]. This sex-specific difference was statistically significant [9]. 

Overall, available studies suggest that sex differences in new cases of stage 3 CKD may vary across populations; however, reported patterns are mixed and highly sensitive to CKD definitions and measurement [1-3,5,8]. A clear synthesis of these sex-specific findings and their methodological limitations remains lacking. For this reason, this review integrates available evidence on sex-based differences in progression to incident stage 3 CKD, using an eGFR less than 60 mL/min/1.73 m² as the most consistent and common threshold across studies.

## Review

Conceptual framework

*KDIGO CKD Staging* 

The KDIGO guidelines recommend classifying CKD using the CGA framework, which incorporates Cause, GFR, and albuminuria categories [4,5]. They emphasize the combined use of glomerular filtration rate (GFR) and urine albumin-to-creatinine ratio (uACR) for risk stratification, highlighting that these two domains are central to staging disease and guiding individualized risk prediction, particularly in the context of emerging therapies [4,5]. Additionally, the guidelines underscore the importance of assessing and monitoring uACR alongside GFR, noting that despite being a simple and clinically valuable test, it is measured in only about one-third of population-based studies globally [4,5]. 

Defining Progression and Incident Stage 3 CKD

Most cohort studies do not explicitly measure transitions between CKD stages. Therefore, an incident eGFR less than 60 mL/min/1.73 m² is used as the most consistently defined operational definition regarding progression to CKD stage 3 [10]. The KDIGO guidelines state, “In adults at risk for CKD, we recommend using creatinine-based estimated glomerular filtration rate” [4]. If cystatin C measurements are found, they may be used to estimate the GFR category using equations that incorporate both creatinine and cystatin C (creatinine and cystatin C-based estimated glomerular filtration rate) [4]. This recommendation relies upon evidence that equations incorporating both creatinine and cystatin C are more accurate and more closely approximate the gold standard measurement of GFR than equations using either marker alone [4].

KDIGO defines CKD based on abnormalities that persist for at least three months [4,5]. However, many cohort studies define incident CKD using a single eGFR threshold, which may lead to misclassification [3]. A single reduced eGFR may reflect an acute or transient decline in kidney function rather than persistent CKD, particularly when repeat measurements at least three months apart are unavailable [4]. This limitation is particularly relevant for sex-stratified analyses, as kidney function estimates vary by equation used [11]. Because serum creatinine is influenced by muscle mass and creatinine production, which differ on average between men and women, reliance on creatinine-based eGFR may contribute to apparent sex differences in CKD incidence and classification [4,5]. As noted, CKD incidence in earlier studies was measured using only albuminuria or older approaches for assessing GFR, including the changes in the reciprocal serum creatinine, the Cockcroft-Gault equation, or the MDRD equation [5,7]. These methods are generally not as accurate as the Chronic Kidney Disease-Epidemiology (CKD-EPI) equations, particularly for individuals not diagnosed with CKD, women, and older adults [7]. In most studies, new-onset CKD was defined as based on eGFR < 60 mL/min/1.73m², sometimes in combination with albuminuria thresholds [4,7]. However, incomplete urinary albumin testing may lead to under recognition of CKD, particularly among individuals with preserved eGFR but clinically significant albuminuria [4,5]. 

Methods

This study is a narrative literature review examining the differences in CKD progression in men and women, with particular emphasis on stage 3 CKD, understood to be an eGFR less than 60 mL/min/1.73 m².

We searched PubMed for studies addressing CKD development and sex differences, using keywords such as “stage 3 CKD,” “eGFR <60,” “CKD progression,” “sex differences,” and “gender differences.” Targeted searches were also conducted for cohort studies on incident-stage 3 CKD with sex-specific outcomes or risk estimates, using terms including “incident stage 3,” “new-onset CKD,” “hazard ratio,” “Cox regression,” “male,” and “female.” Boolean operators (AND/OR) were used to combine search terms.

We included studies published in PubMed from inception through January 2026. Eligible studies were observational cohort studies in adult human populations that examined new cases of CKD defined as eGFR less than 60 mL/min/1.73 m² and reported results by sex or provided sex-specific risks, such as hazard ratios, odds ratios, or relative risks. Studies were excluded if they involved children, individuals on dialysis, kidney transplant recipients, pregnancy-specific cohorts, or did not follow participants longitudinally.

We initially screened articles by titles and abstracts and then conducted full-text reviews when necessary. The reference lists of relevant articles were then examined to locate further eligible articles. Because this was a narrative review, the search was intended to identify representative and clinically relevant studies rather than to ensure exhaustive retrieval of all published literature. Studies were selected based on their direct relevance to incident stage 3 CKD, availability of sex-stratified findings or sex-specific risk estimates, and applicability to adult populations. No eligible studies identified through this search were intentionally excluded; however, relevant studies may have been missed because only PubMed and reference-list screening were used. We summarized the main findings of patterns and risk factors associated with sex-specific CKD development and progression. Five studies met the inclusion criteria and were included in the final narrative synthesis. Article screening and selection were conducted by the authors. Because this study is a narrative review, we did not conduct a formal risk-of-bias assessment or adhere to PRISMA guidelines.

Results

The included studies evaluating sex differences in incident stage 3 CKD (eGFR <60 mL/min/1.73 m²) demonstrated heterogeneous findings influenced by population characteristics, outcome definitions, and analytical approaches [1,2,11]. Key characteristics, definitions, and sex-specific findings across these studies are summarized in Table 1.

**Table 1:** Comparative Studies on Sex Differences in Incident Stage 3 CKD (eGFR <60 mL/min/1.73 m²) CKD: Chronic kidney disease; eGFR: estimated glomerular filtration rate; TyG: triglyceride-glucose; aHR: adjusted hazard ratio; SHR: subhazard ratio

Study (Year, Country)	Sample Size (% Women)	Population Characteristics	CKD Definition	Follow-up Duration	Key Findings
Yu et al. (2015, USA) [7]	1,464 (52.0%)	Primary care patients with diabetes and normal baseline kidney function	eGFR<60 mL/min/1.73 m² (CKD-EPI) or sex-specific microalbuminuria (UACR ≥25 mg/g for women, ≥17 mg/g for men)	Median 3.1 years	Women had a higher risk of incident CKD (SHR 1.37, 95% CI 1.17-1.60); the difference was driven by incident eGFR<60 (116.2 vs 106.5 per 1,000 patient-years)
Miyakoshi et al. (2017, Japan) [8]	171,536 (% not specified)	Longitudinal health screening cohort without baseline CKD	eGFR<60 mL/min/1.73 m² or positive dipstick proteinuria	Median 6.2 years	Male sex was an independent predictor of CKD development in multivariable analysis (sex-specific results not provided)
Lee et al. (2023, South Korea) [1]	11,495,668 (% not specified)	General population with baseline eGFR ≥90 mL/min/1.73 m² and no proteinuria	eGFR<60 mL/min/1.73 m²	Up to eight years	CKD incidence: 208.2 per 100,000 person-years in men, 365.1 in women; sex-specific risk factors identified (diabetes aHR 1.38 in men, 1.29 in women)
Park et al. (2019, South Korea) [2]	14,190,878 (% not specified)	General population with baseline eGFR ≥60 mL/min/1.73 m² and no proteinuria	Incident low eGFR (<60 mL/min/1.73 m²) or incident proteinuria (≥1+ dipstick)	2009-2016	Sex differences are most pronounced for proteinuria: J-shaped association in men (protective 10 g/day alcohol), positive association in women; inverse association with low eGFR in both sexes
Kim et al. (2025, South Korea) [9]	353,140 (% not specified)	Young adults (18-49 years) with ≥2 health screenings	eGFR<60 mL/min/1.73 m² or proteinuria	Median 6-7 years	Increasing TyG index predicted CKD in men (HR 1.22, 95% CI 1.16-1.28 for the highest change group) but not women

Across the five included studies, the direction of sex-specific risk varied by population and outcome definition [1,2,7-9]. Higher incident CKD risk or higher rates of low eGFR were reported in women in the diabetes cohort and one large general-population cohort, whereas male sex was identified as an independent predictor in another cohort [1,7,8]. Other findings were exposure-specific, including an association between increasing TyG index and incident CKD in men but not women, while sex differences related to alcohol use were more pronounced for proteinuria than for low eGFR [2,9]. 

Studies Directly Evaluating Sex Differences in Incident Stage 3 CKD (eGFR < 60)

Yu et al. studied whether sex was associated with new CKD in a prospective primary care cohort of 1,464 diabetes patients with normal baseline kidney function in Seattle, Washington [7]. Women made up 52.0% of the cohort [7]. They defined CKD as the first time a patient had either eGFR <60 mL/min/1.73 m² (CKD-EPI) or microalbuminuria, using different urine albumin-to-creatinine ratio thresholds for men and women (uACR ≥25 mg/g for women and ≥17 mg/g for men) [7]. The study also considered mortality as a competing event to reduce survival bias, and found that men had higher mortality than women [7].

The analysis showed that women had an increased risk of developing CKD, with a subhazard ratio (SHR) of 1.37 (95% CI 1.17-1.60), and this remained after further adjustment (SHR 1.35, 95% CI 1.15-1.59) [7]. This difference between men and women was seen across age groups and was mostly due to new cases of low eGFR, rather than microalbuminuria [7]. In adjusted models, women continued to have a higher risk of low eGFR (e.g., SHR 1.51, 95% CI 1.24-1.84) [7]. The rate of eGFR <60 was 116.2 events per 1,000 patient-years in women and 106.5 per 1,000 patient-years in men [7]. The models accounted for demographics, baseline eGFR, diabetes duration, and clinical CKD risk factors [7]. The results remained consistent across sensitivity analyses limited to people with type 2 diabetes and those with complete baseline uACR results [7]. 

Longitudinal Cohort Studies Identifying Sex as a Predictor of Incident CKD

Miyakoshi et al. examined which factors predicted new cases of CKD in a longitudinal health screening cohort of 171,536 adults who did not have CKD at baseline and were re-examined after a median follow-up of 6.2 years [8]. CKD was defined as eGFR <60 mL/min/1.73 m² or positive dipstick proteinuria [8]. Their analysis found that male sex, older age, higher systolic blood pressure, higher uric acid concentration, and lower baseline eGFR all increased the risk of developing CKD [8]. The strongest predictor was baseline kidney function; for example, participants with baseline eGFR <70 mL/min/1.73 m² had an adjusted hazard ratio (aHR) of 90.1 [8]. This study did not provide separate results for men and women, but did find that male sex was an independent predictor in multivariable modeling [8].

Sex-Specific Associations and Effect Modification in Large Population Cohorts

Several large population-based cohorts examined how sex affects the risk of eGFR <60 and whether there are any interaction effects. Park et al. examined whether alcohol intake was associated with incident CKD outcomes and whether these associations differed by sex [2]. Researchers analyzed health screening data from 14,190,878 adults who underwent ≥3 screenings between 2009 and 2016 and had baseline eGFR ≥60 mL/min/1.73 m² with a negative urine dipstick for proteinuria [2]. Incident low eGFR was defined as eGFR <60 mL/min/1.73 m² during follow-up, and incident proteinuria was defined as dipstick proteinuria ≥1+ during follow-up [2]. Sex-specific patterns differed most clearly for proteinuria: modest alcohol intake (<10 g/day) was associated with lower incident proteinuria risk in men, with a J-shaped pattern, whereas women did not demonstrate a similar protective association and showed increased proteinuria risk across intake levels [2]. In contrast, alcohol intake was associated with a smaller annual decline in eGFR in both sexes and with a lower risk of incident low eGFR in both men and women [2].

Lee et al. developed risk prediction models for CKD specific to men and women, using data from the South Korean National Health Insurance Service registry database [1]. Their cohort included 11,495,668 adults who started with baseline eGFR ≥90 mL/min/1.73 m² and no proteinuria, and who had ≥2 health screenings between 2009 and 2016 [1]. CKD was defined as eGFR <60 mL/min/1.73 m² during follow-up [1]. The rate of new CKD cases per 100,000 person-years was 208.2 for men and 365.1 for women [1]. Diabetes was one of the strongest risk factors for both sexes (aHR 1.38, 95% CI 1.34-1.41 in men; aHR 1.29, 95% CI 1.26-1.32 in women) [1]. Some risk factors differed for men and women, including waist circumference, past smoking in men, and treatment history of heart disease in women [1].

Kim et al. studied a cohort of 353,140 Korean adults between 18 and 49 years of age who completed a minimum of two health screenings [9]. They defined new CKD as eGFR <60 mL/min/1.73 m² or proteinuria [9]. The median follow-up was approximately six to seven years for both men and women [9]. Their analysis showed that men in the largest TyG index change group had a higher risk of CKD incidence (HR 1.22, 95% CI 1.16-1.28), while those in the lowest group had a lower risk (HR 0.88, 95% CI 0.84-0.92) compared to the reference group [9]. For women, these hazard ratios were not statistically significant [9]. The association between TyG index change and sex was statistically significant (p-interaction <0.001), showing that the TyG-CKD link was different for men and women [9]. The association was strongest among adults aged 40-49 years, those with baseline eGFR <90 mL/min/1.73 m², those with higher alcohol intake (≥20 g/day), and those in the highest homeostatic model assessment for insulin resistance (HOMA-IR) quartile [9]. In this study of young adults, a rising TyG index over time was linked to higher incident CKD risk in men but not in women [9]. 

Discussion

This review aims to synthesize current evidence on sex differences in the development of stage 3 CKD, using incident eGFR <60 mL/min/1.73 m² as the main reported operational definition. The available studies show substantial variation, and most define incident CKD cases using a single eGFR threshold rather than tracking changes over time [1,2,9,11]. However, both studies involving people with diabetes and the general population often found sex differences or sex-specific links in CKD outcomes, though the patterns change depending on population, exposure, and how outcomes were defined [1,2,7,8,11]. Overall, these results suggest that sex differences in incident stage 3 CKD may be present, but they depend on how CKD is defined and measured [1-3,5,7,8]. The KDIGO 2024 guidelines state that "progression of CKD has been reported as more rapid in men, in women, or no difference by sex or gender," and they attribute these inconsistencies to differences in disease cause and outcome definitions [4,5]. Women have a higher prevalence of CKD (11.8% compared to 10.4% in men), but men tend to have a faster eGFR decline and are more likely to develop end-stage kidney disease [12]. These differences illustrate the complexity of sex-specific CKD patterns.

Several biological and metabolic mechanisms may contribute to the observed sex-specific patterns in CKD development. Differences in body composition and muscle mass can influence serum creatinine production, and consequently, creatinine-based eGFR estimates, potentially affecting CKD classification in men and women [4,5]. Sex-related biological factors may also influence CKD prevalence and progression, although the direction of these effects is not consistent across populations [12]. Metabolic factors may further contribute, as longitudinal increases in the TyG index were associated with incident CKD in men but not women in one included cohort [9]. These biological and metabolic influences likely interact with differences in study populations, CKD definitions, and measurement methods rather than producing one uniform sex-specific pattern [4,5,9,12]. 

Yu et al. found that in a cohort of primary care patients with diabetes and normal kidney function, women had a higher likelihood than men to develop CKD after a median follow-up of 3.1 years, even when adjusting for mortality as a competing event [7]. Yu et al. pointed out that earlier studies in diabetic cohorts often reported higher CKD risk in men, but many account for mortality as a competing risk, which is important because life expectancy among men with diabetes is typically lower than in women [7]. The sex difference in new CKD cases was mainly due to differences in incident eGFR < 60 mL/min per 1.73 m²; while there was a trend toward higher risk for microalbuminuria in women, this was not statistically significant [7]. These results suggest that both biological factors and study methods may affect sex differences in diabetic kidney outcomes, and that the use of competing-risk models can alter the noted patterns [7]. The protective effect of female sex observed in general populations appears diminished in diabetes, where metabolic dysregulation may override hormonal protection [13]. Overall, the authors concluded that women with mostly type 2 diabetes had a higher risk of developing CKD that could not be fully accounted for even after adjusting for traditional CKD risk factors, depression, or diabetes self-care behaviors and called attention to the need for more research to understand the causes and long-term effects [7].

Miyakoshi et al. also identified male sex was an independent predictor of developing CKD in multivariable analysis, along with age, blood pressure, uric acid, and baseline eGFR [8]. Although they did not provide separate effect estimates by sex, this finding supports the inclusion of sex as a predictor in CKD risk models [8].

Large studies in the general population provide additional evidence that sex can modify CKD risk relationships [1]. Lee et al. developed separate risk prediction equations for men and women to identify new CKD cases among over 11 million adults with normal kidney function and no protein in the urine at baseline [1]. Diabetes was a strong predictor for both sexes, but some risk factors were different: waist circumference and past smoking mattered more in men, while a history of heart disease treatment was important for women [1]. This evidence supports that the risk profiles for developing CKD can differ by sex, even when using the same eGFR threshold [1].

Park et al. also looked at alcohol use and new CKD cases in over 14 million adults and identified sex-specific patterns, especially for proteinuria [2]. Studies on the link between alcohol and new cases of CKD have shown mixed results, including inverse, positive, and J-shaped associations [2]. There has been little research on sex differences in this relationship, and a few existing studies have yielded inconsistent results [2]. In men, drinking less than 10 grams of alcohol per day was linked to a lower risk of proteinuria, but women did not have this benefit and instead showed increased proteinuria risk at all alcohol intake levels [2]. For outcomes based on kidney filtration (such as annual eGFR decline and new low eGFR), both sexes demonstrated an inverse relationship with alcohol intake in both sexes [2]. Although the reasons for the sex differences in how alcohol intake relates to the risk of developing proteinuria are still unclear, these results suggest that sex differences depend on whether CKD is measured by proteinuria or eGFR, supporting the need to look for both when studying sex-specific CKD risk [2].

New research also supports that metabolic factors linked to incident CKD may differ by sex. Kim et al. studied changes over time in the TyG index, a marker of insulin resistance, specifically in young adults [9]. They found that a rising TyG level predicted new cases of CKD in men but not in women. The interaction between TyG changes and sex was statistically significant in adjusted models (p-interaction <0.001), indicating a sex-specific effect. Subgroup analyses examined whether this association varied by age, baseline eGFR, alcohol use, and severity of insulin resistance (HOMA-IR). The TyG-CKD link was strongest in adults aged 40-49, those with lower baseline eGFR <90 mL/min/1.73 m², higher alcohol intake (≥20 g/day), and the highest HOMA-IR group [9]. The authors noted that the study had ≥95% power to detect the observed effects in men, but less so in women, because the observed hazard ratios were close to 1 [9]. They suggested that this male-specific link may be due to sex differences in metabolic risk, hormones, and body fat, which could modify how insulin resistance affects kidney function [9]. Overall, these results indicate that insulin resistance-related kidney risk may differ between men and women, particularly in younger people [9,14].

Methodological issues and measurement bias in sex-stratified CKD research

One major limitation across the included studies is reliance on creatinine-based eGFR instead of directly measuring GFR. KDIGO recommends using the CGA classification system and combining GFR with the uACR to stage and predict risk [4,5]. KDIGO also notes that biological factors affecting creatinine or cystatin C can alter eGFR results [4,5]. KDIGO further notes that when cystatin C is available, using equations that include both creatinine and cystatin C yield results closer to measured GFR and improve accuracy, particularly when creatinine-based estimates may be unreliable [4,5]. This methodological point is important in sex-stratified studies, since creatinine-based eGFR can be affected by differences in muscle mass and creatinine production between men and women [4]. 

Many studies define new CKD cases based on a single eGFR reading below 60, which can lead to misclassification compared to KDIGO’s rule, which requires abnormalities to persist for at least three months [4,5]. Checking for albuminuria is often incomplete, and studies use different methods to measure urinary protein, such as uACR or dipstick tests, making it hard to compare results across cohorts and potentially affecting sex-specific findings [2]. In the study by Yu et al., not all participants had baseline uACR tests, so some individuals might have been misclassified as having normal kidney function [7]. However, sensitivity analyses in those with baseline uACR yielded results similar to those of the main analysis [7]. Yu et al. also noted that women had more uACR tests than men, which could make CKD detection more likely among women and may help explain the observed sex differences [7]. Regardless of these issues, Yu et al. had several methodological strengths, including prospective follow-up, competing-risk modeling for mortality, clear albuminuria thresholds, and adjustment for multiple CKD risk factors [7]. 

Additional limitations affecting interpretation and generalizability

There is still a risk of confounding because many lifestyle factors are self-reported, diet is not adequately adjusted for, and some studies do not account for kidney-protective medications [9]. Also, generalizability may be limited as several studies used Korean national screening data that may not apply to populations with different ethnic backgrounds or lifestyles [1,2]. KDIGO also points out that many studies do not fully consider CKD cause, sex versus gender, age, socioeconomic status, or uACR, making it harder to interpret and compare results across cohorts [4]. In addition, some studies examined only baseline exposures, which may not reflect long-term risk patterns [9]. Overall, differences in outcome definitions, incomplete albuminuria data, and measurement bias may explain why sex differences are reported across studies [1,2,4]. These limitations demonstrate why it is hard to interpret sex-stratified CKD progression and reinforce the importance of standardized outcome definitions in future research. Together, differential testing practices, residual confounding, and variation in CKD definitions may contribute to the inconsistent direction and magnitude of sex-specific associations reported across studies [1,2,7,9]. 

## Conclusions

Substantial variation remains in the evidence regarding the progression of early CKD to stage 3 in men and women, with inconsistent patterns reported across studies. These differences likely reflect a combination of biological, clinical, and measurement-related factors rather than a single uniform pattern. Potential explanations include biological variation, differences in lifestyle and metabolic exposures, disparities in mortality, and limitations in how kidney function is measured. Outcome definitions, including reliance on eGFR thresholds alone or the inclusion of albuminuria, may influence how sex differences are observed and interpreted.

For clinical practice, these findings emphasize the importance of regular monitoring of kidney function and albuminuria in both sexes, particularly among individuals with diabetes or metabolic risk factors. Future research should prioritize consistent definitions, repeated measurements, and more comprehensive assessment of kidney function to better understand sex-specific risk and progression to stage 3 CKD. Clinically, routine albuminuria testing and confirmation of reduced eGFR with repeated measurements should be emphasized in both sexes, while future risk-prediction models should evaluate whether incorporating sex-specific factors improves identification of individuals at risk for stage 3 CKD. 
